# Cyclooxygenase-2 (COX-2) inhibitors: future therapeutic strategies for epilepsy management

**DOI:** 10.1186/s12974-019-1592-3

**Published:** 2019-10-30

**Authors:** Chitra Rawat, Samiksha Kukal, Ujjwal Ranjan Dahiya, Ritushree Kukreti

**Affiliations:** 1 Genomics and Molecular Medicine Unit, Institute of Genomics and Integrative Biology (IGIB), Council of Scientific and Industrial Research (CSIR), Mall Road, Delhi, 110007 India; 2 Academy of Scientific and Innovative Research (AcSIR), Council of Scientific and Industrial Research (CSIR), Delhi, India

**Keywords:** Cyclooxygenase-2 (COX-2), Seizure, Epilepsy, Inflammation, Blood-brain barrier (BBB), Anticonvulsant, Adjunctive

## Abstract

Epilepsy, a common multifactorial neurological disease, affects about 69 million people worldwide constituting nearly 1% of the world population. Despite decades of extensive research on understanding its underlying mechanism and developing the pharmacological treatment, very little is known about the biological alterations leading to epileptogenesis. Due to this gap, the currently available antiepileptic drug therapy is symptomatic in nature and is ineffective in 30% of the cases. Mounting evidences revealed the pathophysiological role of neuroinflammation in epilepsy which has shifted the focus of epilepsy researchers towards the development of neuroinflammation-targeted therapeutics for epilepsy management. Markedly increased expression of key inflammatory mediators in the brain and blood-brain barrier may affect neuronal function and excitability and thus may increase seizure susceptibility in preclinical and clinical settings. Cyclooxygenase-2 (COX-2), an enzyme synthesizing the proinflammatory mediators, prostaglandins, has widely been reported to be induced during seizures and is considered to be a potential neurotherapeutic target for epilepsy management. However, the efficacy of such therapy involving COX-2 inhibition depends on various factors viz., therapeutic dose, time of administration, treatment duration, and selectivity of COX-2 inhibitors. This article reviews the preclinical and clinical evidences supporting the role of COX-2 in seizure-associated neuroinflammation in epilepsy and the potential clinical use of COX-2 inhibitors as a future strategy for epilepsy treatment.

## Background

Epilepsy, a common neurological disorder, is characterized by (1) at least two unprovoked seizures occurring > 24 h apart, (2) one unprovoked seizure and a probability of further seizures similar to the general recurrence risk (at least 60%) after two unprovoked seizures, occurring over the next 10 years, or (3) diagnosis of an epilepsy syndrome [1]. The underlying pathophysiology behind the disease progression is still unknown, and therefore, the only available remedy is to control the frequency of seizures using the symptomatic treatment, antiepileptic drugs (AEDs). However, nearly 30% of the patients do not respond to the available AEDs [2], emphasizing the need to develop better effective therapies which can target epileptogenesis.

Investigations to elucidate the mechanisms involved in epileptogenesis provided strong evidences on the crucial role of inflammation as the cause as well as consequence of seizure and epilepsy development [3]. In this regard, inflammatory molecules such as cytokines, chemokines, and prostaglandins (PGs) are often observed to be released by the brain and brain capillary endothelial cells affecting neuronal function and excitability in preclinical and clinical set-ups [4]. Blocking the undesired inflammatory signaling using anti-inflammatory molecules may provide novel strategies to treat epilepsy [5]. The inflammatory molecules might, therefore, serve as therapeutic targets to develop better effective medications for epilepsy management.

Over the past two decades, cyclooxygenase-2 (COX-2), being the central link to various inflammatory processes, has received much attention due to its involvement in seizure generation and epilepsy development. COX-2 has been reported to be upregulated in different cells within the brain following seizure induction leading to increased production of proinflammatory mediators, PGs, which further aggravates seizure severity [6]. Besides, in vivo evidences suggest that COX-2 induction following seizure may upregulate the multidrug efflux transporter P-glycoprotein (P-gp) at the blood-brain barrier (BBB) causing reduced delivery of administered AEDs to the brain target site, thus leading to poor efficacy [7]. Such studies proposed that inhibition of COX-2, genetic or pharmacological, might reduce seizure severity and pharmacoresistance to AEDs and thus could be exploited as a future strategy for epilepsy treatment.

In this article, we review the preclinical and clinical evidences supporting the role of COX-2 in seizure-associated neuroinflammation in epilepsy and its regulatory effect governing patient’s response to AEDs. This review also addresses the potential therapeutic use of COX-2 inhibitors as (1) anticonvulsants for epilepsy management or (2) adjunctives to AED therapy to overcome pharmacoresistance.

### Inflammation in epilepsy

Neuroinflammation comprises activation of microglia, astrocytes, brain capillary endothelial cells, and circulating peripheral immune cells along with the production of inflammatory mediators, initiated in response to a variety of stimulus such as traumatic brain injury, brain infection, and autoimmunity. Evidences revealing the altered expression of different cytokines, chemokines, and other immune-related molecules in epilepsy indicated inflammation as a crucial factor contributing in its pathogenesis. Increased glial cellular expression of a cytokine IL-1α was observed in patients with drug-resistant temporal lobe epilepsy (DRTLE) with complex partial seizures [8]. An equally potent inflammatory cytokine *IL1B* was also found to be associated with epilepsy when a polymorphism in that gene responsible for increasing the production of IL-1β was detected in patients with temporal lobe epilepsy (TLE) [9]. Besides, cerebrospinal fluid (CSF) as well as serum samples from patients experiencing seizures exhibited increased levels of different cytokines such as IL-1β, IL-6, IL-1Ra, and IFNγ, substantiating the role of such cytokines in seizure sustenance [10–13]. A recent follow-up study compared central and peripheral levels of IL-1β and IL-6 in sera of drug-resistant patients before surgical treatment and 1 year after surgery when most patients were either seizure-free or had reduced seizures [14]. The respective levels were found to decrease in the absence of seizures after surgical treatment indicating seizure-induced inflammation. Gene expression profiling of surgically removed hippocampal tissue from patients with TLE revealed upregulation of several chemokines, CCL2, CCL3, and CCL4 along with the chemokine receptor, CXCR4 [15]. The chemokine ligand CX3CL1 was also observed to be upregulated in the hippocampus and the adjacent cortex of epileptic rats as well as in temporal neocortex of patients with TLE [16]. CX3CL1 was further reported to be elevated in the CSF and serum of the same patients compared to the non-epileptic group. Expression of another C-X-C chemokine motif ligand CXCL13 and its receptor CXCR5 were also altered in brain tissues of patients with DRTLE [17]. These alterations were associated with changes in the molecules regulating the cytokines. Severe neuronal loss and persistent overexpression of NFκB-p65, a key regulator of acute inflammatory reactions, was noticed in reactive astrocytes in human medial TLE with hippocampal sclerosis (HS) [18]. The findings were strengthened by Das et al. who revealed an upregulation of NF-κB-p65 along with COX-2 enzyme and TGF-β in the hippocampal region as the key molecular events associated with histopathological changes observed in DRTLE [19]. Such clinical evidences and their similarities with the findings of rodent studies promoted the use of in vivo animal models to determine the putative mechanism underlying the shared link between inflammation and seizures.

Accumulating evidences in in vivo experimental models suggested the role of inflammation as either the cause or the consequence of epilepsy contributing to its pathophysiology [3]. Findings from the studies performed in rodent epilepsy models showed activation of hippocampal astrocytes and glial cells along with the elevation of inflammatory mediators in the hippocampus. A transient time-dependent increase in expression of important inflammatory cytokines IL-1β, IL-6, and TNFα was observed in the hippocampus of electrically induced limbic status epilepticus (SE) rat model [20]. SE induction in mouse altered the expression of chemokine receptors, CCR3 and CCR2A, and their ligands in the brain and thereby may weaken the neuroprotective mechanisms [21]. Furthermore, microarray analysis of different brain regions of rat model of TLE during epileptogenesis indicated alterations in inflammatory molecules such as interleukins in the acute, latent, and chronic phase of epilepsy [22]. Besides the cytokines, induction of the proinflammatory enzyme COX-2 was observed in hippocampal and neocortical neurons upon hippocampal kindling in rats, suggesting COX-2 induction to be a key signaling event in epileptogenesis [23]. A remarkable rise in the production of COX enzyme products, i.e., PGs, was observed along with the increased COX-2 expression, following seizure induction in rodents [6, 24]. Such studies illustrate inflammation as a consequence of seizure induction and epilepsy.

Systemic administration of lipopolysaccharide (LPS), an inducer of inflammation, prior to SE induction increased hippocampal vulnerability to seizure-induced neuronal injury in immature rats, suggesting the involvement of inflammation in seizure etiology [25]. LPS administration in immature rats also increased kindling progression indicating LPS-induced inflammation to enhance epileptogenesis [26]. Administration of high doses of IL-1β, a pyrogenic proinflammatory cytokine, induced febrile seizures in only IL-1β receptor-expressing mice whereas IL-1β receptor-deficient mice were resistant to seizure generation [27]. Brain transcriptome profiling in Wistar rats after epileptogenic treatment revealed TGF-β signaling as a novel inflammatory cascade involved in influencing the generation of epileptiform activity [28]. Endogenous nitric oxide (NO), another proinflammatory mediator, increased seizure activity in mice brain slices and reported as a key promoting factor for initiation of seizure-like events [29]. Similarly, intrahippocampal injection of HMGB1, a cytokine-like proinflammatory molecule, increased seizure frequency in epileptic rats [30]. However, the effect abrogated in case of TLR-4 mutant mice, recognizing the involvement of HMGB1-TLR4 inflammatory axis in generating seizures. Furthermore, expression of different inflammatory mediators TLR4, ATF-3, and IL-8 in the epileptic brain tissue of patients with mesial TLE correlated with their seizure frequency, supporting the etiologic role of inflammation in seizure generation [31]. Therefore, inflammation plays a reciprocal role in epilepsy, i.e., an outcome of seizure activity as well as a contributing factor in the disease development.

### Inflammation: a potential therapeutic target

Current anticonvulsive therapy primarily consists of AEDs which only control the frequency of seizures and hence considered symptomatic. AEDs function by exerting a number of concurrent mechanisms involving both the excitatory and inhibitory synapses. Common modes of action include targeting ion channels and neuroreceptors embedded in the cell membrane to regulate neuronal excitability. Most of the AEDs such as phenytoin, carbamazepine, valproate, and topiramate act as sodium channel blockers by stabilizing the inactivated state of these channels, thus preventing the neuronal depolarization and excitability [32]. On the contrary, activation of potassium channels by retigabine causes a generalized reduction in neuronal excitability by driving the membrane potential to a hyperpolarized state [33]. Gabapentin and pregabalin act on pre-synaptic calcium channels and blocks calcium-mediated release of neurotransmitter across the synapse, preventing the synaptic conduction [34]. Simultaneously, inhibition of glutamate receptor by topiramate prevents the effect of the excitatory neurotransmitter, glutamate, while agonists of the inhibitory neurotransmitter, γ-aminobutyric acid (GABA), such as benzodiazepines activate GABA receptor preventing generation of neuronal action potential. Valproate and vigabatrin increase GABA turnover by blocking its degradation [32, 33]. Levetiracetam works by binding to the synaptic vesicle protein, SV2A, causing a reduction in the vesicular release of neurotransmitter, thus differing in its mechanism from other AEDs [34].

Despite the availability of a wide spectrum of AEDs with diverse pharmacological targets, 30% of patients experience multidrug resistance in epilepsy [35]. Various hypotheses have been proposed to address the molecular mechanism behind this drug resistance. Drug target hypothesis suggested that resistance to AEDs is caused by genetic or acquired alterations at their target sites (ion channels or neuroreceptors associated with neuronal excitability) affecting the pharmacodynamics of the drug [36]. Genetic polymorphisms in the target genes such as SCN1A and GABRA1 have been observed to be associated with AED dose requirements or AED resistance [37–40]. Transporter hypothesis proposed that increased expression of ATP-binding cassette (ABC) efflux transporters at the BBB interferes with the pharmacokinetics of the AEDs leading to their decreased concentration at the target site [41]. Brain endothelial cells and other brain cells of patients with refractory epilepsy have been reported to have increased expression of ABC transporters compared to healthy individuals [42–44]. A third recent hypothesis known as intrinsic severity hypothesis correlated AED resistance with the severity of the disease [45]. The higher the seizure frequency, the more difficult it is to treat with the available AEDs [46, 47]. Another recent hypothesis called the methylation hypothesis proposed that seizures can mediate epigenetic modifications that result in persistent genomic methylation, histone density, and posttranslational modifications, as well as noncoding RNA-based changes leading to pharmacoresistance in epilepsy [48]**.**

In view of the above hypotheses, till date, nothing conclusive has been gained to address pharmacoresistance in epilepsy, suggesting a gap in the current pharmacological research which often overlooks inflammation as a primary therapeutic target in managing epilepsy. Findings of studies examining whether the currently available AEDs possess anti-inflammatory action are quite debatable. Levetiracetam displayed anti-inflammatory property by reducing immunoreactivity of astrocytic and glial IL-1β system in epileptic rat hippocampus; on the other hand, valproate was unable to show such effects [49]. However, whether the drug has primary anti-inflammatory properties or secondary to reduced seizure activity is still a question. Pre-administration of vinpocetine and carbamazepine before LPS also reduced the brain mRNA levels of IL-1β and TNF-α in rats while valproate again showed neutral behavior [50]. The same study also showed complete prevention of seizure activity along with decreased IL-1β and TNF-α mRNA levels in the groups pre-administered with the two AEDs before the pro-convulsive drug, 4-aminopyridine, suggesting that the anti-inflammatory action of these drugs might be achieved primary to reduced epileptic activity. Further investigation on such evidences is required to better understand the mechanism of anticonvulsive agents. Contrary to these findings, Verotti et al. observed that both carbamazepine and valproate induced inflammation by increasing interleukin and chemokine levels in children with epilepsy on 1 year of monotherapy [51]. The conflicting findings on the effect of these AEDs on inflammation hint towards the need of a better efficacious anti-inflammatory treatment to manage epilepsy.

Given the indispensable role of inflammation in epilepsy pathogenesis, inflammatory molecules may be considered as important therapeutic targets for epilepsy management; however, evidence on the efficacy of such therapy is limited. Vezzani et al. demonstrated powerful anticonvulsant action of IL-1Ra, an endogenous IL-1 receptor antagonist, by blocking the proinflammatory action of IL-1β in mice [52]. Another study observed a delay in onset of seizure and decrease in its duration when the administered caspase-1 inhibitor reduced IL-1β levels in SE rats [53]. The similar finding was observed in a more recent study, where epigenetic impairment of IL-1β/TLR4 pathway reduced seizure frequency by approximately 50% while the widely prescribed AED, carbamazepine, was ineffective, again representing the anti-inflammatory therapy as a pivotal strategy to manage drug-resistant epilepsy [54]. Selective PG EP2 receptor antagonist also reduced SE-induced neuronal injury by preventing COX-2 upregulation in rats [55]. Supporting this, administration of rofecoxib, a COX-2 inhibitor, potentiates the anticonvulsant activity of subeffective dose of tiagabine against pentylenetetrazol (PTZ)-induced convulsions in mice [56]. Therefore, inhibition of such inflammatory molecules may serve as an effective treatment strategy for drug-resistant epilepsy.

**Fig. 1:**
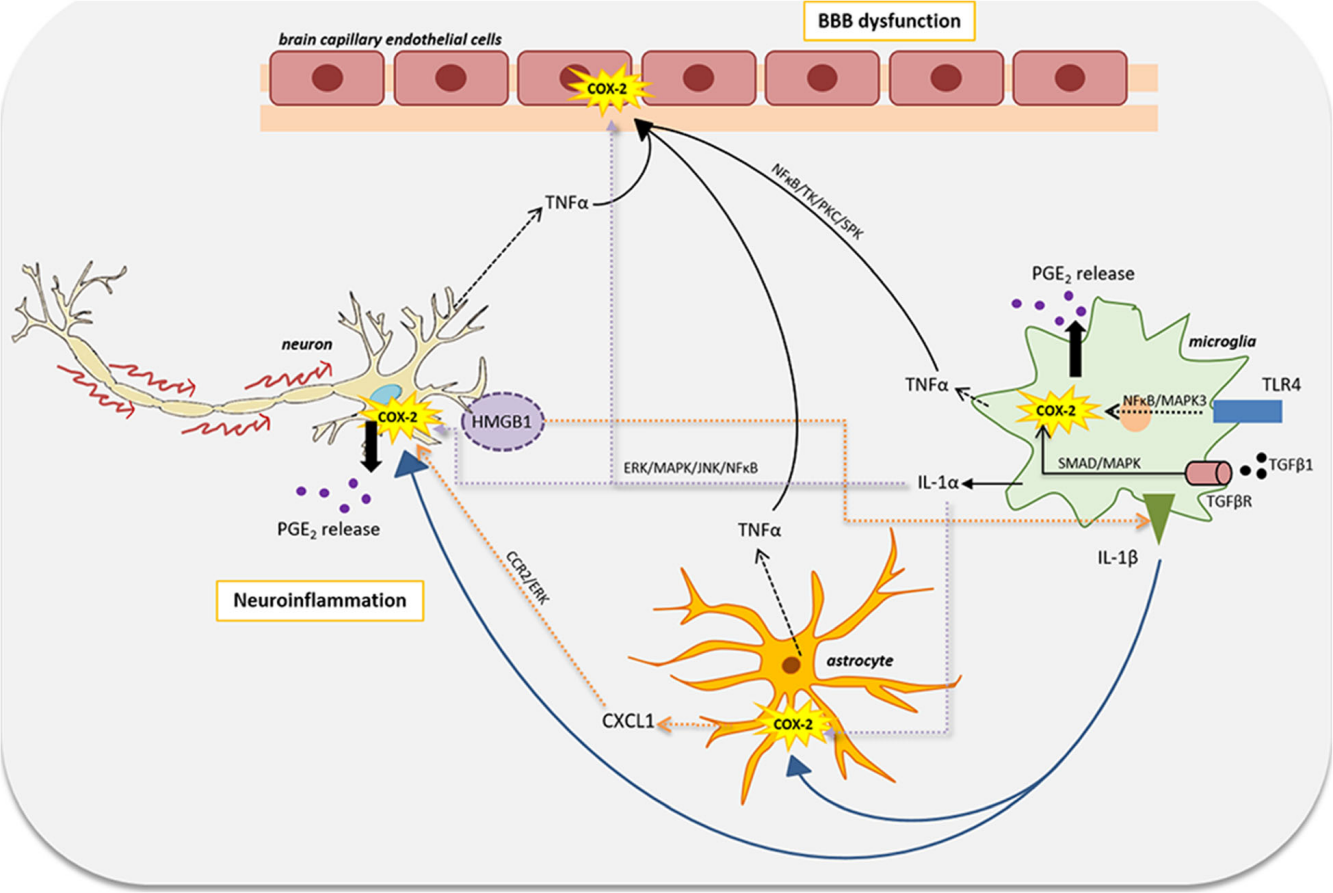
Regulatory pathways linking COX-2 with pro-inflammatory cascades in the brain: epilepsy induces neuroinflammation as well as peripheral inflammation which further reciprocate by aggravating the disease. Several pro-inflammatory processes involving the cytokines, chemokines, toll-like receptors, etc. often cause induction of the enzyme, COX-2, which increases the production of the lipid mediators, prostaglandins (PGs), majorly PGE_2_. COX-2 induction in the brain capillary endothelial cells can cause blood-brain barrier (BBB) dysfunctioning leading to enhanced efflux of the administered AEDs and therefore, may lower their delivery to brain resulting in reduced AED efficacy. Activation of microglia and astrocytes may also result in COX-2 induction contributing to the build-up of PGE_2_ and other inflammatory mediators in themselves as well as neurons, thereby causing neuroinflammation

Inflammation is a complex biological process involving several proinflammatory as well as anti-inflammatory mechanisms. While application of a polypharmaceutical approach may have unintended consequences through drug-drug interactions [57], targeting an inflammatory cascade which involves both pro- and anti-inflammatory molecules may be exactly what would produce the desired effects by reducing inflammation, provided the anti-inflammatory mechanisms remain intact. Therefore, identification of such cascade involving different inflammatory processes is essential to produce broad-spectrum efficacious treatment. Notably, most of the published findings on crosstalk or signaling pathways of inflammation converge at the common proinflammatory gene, COX-2 (Fig. 1). COX-2 is rapidly induced after a pro-inflammatory event with a subsequent release of PGs, potent mediators of inflammatory responses. IL-1β released by activated microglia during inflammation of the central nervous system (CNS) was shown to induce COX-2 and biosynthesis of its proinflammatory product, PGE_2_, in mice astrocytes [58] and in human neuroblastoma cells [59]. Increased levels of HMGB1 found within inflamed synovium of rheumatoid arthritis patients potentiate IL-1β to stimulate COX-2 and prostanoid synthesis [60]. Similarly, the proinflammatory cytokine, TNFα, also induces COX-2 expression and PGE_2_ release resulting in enhanced vascular permeability and cytoskeletal changes in brain capillary endothelial cells [61]. The signaling pathways connecting such cytokines to COX-2 expression may involve regulatory kinases such as SPK, TK, PKC, NF-κB, ERK, and MAPK [58, 62–64]. The chemokine, CXCL1, induced COX-2 via ERK, thus mediating astroglial-neuronal interaction to enhance sensitization towards neuropathic pain [65]. More recently, activation of COX-2 and PG production by TGF-β1 via ALK5/SMAD and MEK/ERK pathway in dental pulp cells have been demonstrated to be an early event in tissue inflammation and regeneration [66]. Besides the cytokines, TLR4 was also found to regulate COX-2 in TLR4-positive human intestinal epithelial cells which expressed higher COX-2 levels upon LPS exposure compared to TLR4-negative cells [67]. Simultaneously, in vivo induction of colitis showed increased COX-2 expression in wild-type mice compared to TLR4-deficient mice. Besides, the anti-inflammatory cascades involving IL-10, IL-4, and IL-1Ra have also been shown to regulate COX-2 in different cells [68–70]. Moreover, administration of COX-2 inhibitors, ibuprofen and celecoxib, following traumatic brain injury in rats showed no significant difference in brain IL-10 indicating anti-inflammatory mechanisms to remain intact in presence of COX-2 inhibitors [71]. COX-2, therefore, serves as a downstream molecule to several inflammatory processes. In addition, change in the COX-2 activity itself has also been reported to alter the pro-inflammatory pathways. Treatment with a selective COX-2 inhibitor, SC-58125, reduced synovial inflammation by reducing local and systemic IL-6 levels, thus reversing paw edema in adjuvant-induced arthritis rat model showing regulation of IL-6 by COX-2-derived PGs [72]. Consistent with this, increased PGE_2_ production and IL-6 levels in IL-1β-stimulated human fibroblast-like synoviocytes from patients with disk displacement were reversed on in vitro treatment with COX-2 inhibitors, celecoxib, and indomethacin [73]. Indomethacin also reduced IL-1β and TNF-α expression in hippocampus of pilocarpine-induced SE rat model [74] demonstrating a regulatory effect of COX-2 on these inflammatory molecules. COX-2, therefore, acts as a central signaling molecule for various inflammatory processes and could be explored as a potential therapeutic target for the management of numerous diseases including epilepsy.

### Cyclooxygenases (COX)

Prostaglandin-endoperoxide synthases (PTGS), also known as COX, are enzymes which synthesize prostanoids involving PGs and thromboxane from their substrate arachidonic acid (AA). Mammalian COX enzyme group consists of two widely studied and well-understood oxygenases, COX-1 and COX-2, responsible for the synthesis of the proinflammatory mediators, PGs. With the help of mutagenesis, X-ray crystallography, and kinetic studies, their structure and mechanism of action are well established [75]. The two isoforms COX-1 and COX-2 catalyzing the synthesis of inflammatory molecules are almost similar in weight with 60–65% sequence similarity; however, they differ in their localization and expression. While COX-1 is constitutively expressed in nearly all tissues, COX-2 is an inducible enzyme primarily localized to immune cells such as macrophages and leucocytes and upregulated in pathological conditions [76]. Further, COX-1 is believed to be responsible for homeostatic PG production whereas COX-2 produces PGs which are generally pathophysiological in nature [77]. Exposure to endotoxins, cytokines, and mitogens induces COX-2 in different cell types such as chondrocytes and macrophages, suggesting the involvement of COX-2 in inflammatory responses. In this regard, administration of COX-2 inhibitory drugs is considered as the treatment to suppress inflammation in various acute and chronic conditions. The last two decades have seen tremendous research on the role of COX-2 in various neuroinflammatory diseases including epilepsy and its applicability as a neurotherapeutic target.

### COX-2-mediated neuroinflammation in epilepsy

COX-2-mediated neuroinflammation is a subject of broad and current interest in basic epilepsy research. Yamagata et al., for the first time, demonstrated immediate induction of COX-2 mRNA and protein in rat hippocampus and cerebral cortex following a maximal electroconvulsive seizure [78]. They also demonstrated this seizure-mediated COX-2 induction to be regulated by N-methyl-d-aspartate (NMDA) receptor-dependent synaptic activity. They further proposed that COX-2 induction during seizure leads to the activation of PG signaling pathway, consequently triggering secondary damage to the brain and amplifying disease severity. Thereafter, several studies replicated the findings in other rat brain tissues, striatum, brain stem and cerebellum, besides hippocampus and cerebral cortex in kainic acid (KA) as well as electroconvulsive shock-induced seizures, suggesting delayed neuronal damage of an interconnected neuronal network during COX-2 activation [79–81]. COX-2 induction, in turn, facilitated recurrence of hippocampal seizures in a mouse model of kindling by synthesizing more PGE_2_ [6]. Production of PGE_2_, the major product of COX-2, was observed to be increased along with COX-2 induction following seizures, stimulating neuronal loss in rodent models of epilepsy [82, 83]. Furthermore, combining PGE_2_ with subconvulsant dose of PTZ caused seizures whereas administration of PGE_2_ antibodies attenuated PTZ-induced seizures in rats, supporting a key role of PGE_2_ production in triggering seizure and maintaining its threshold [84]. PGE_2_ binds to G protein-coupled receptors (GPCRs), namely EP1, EP2, EP3, and EP4, with highest affinity for the EP1 receptor. Activation of EP receptors results in increased calcium ion influx which in turn enhances glutamate release presynaptically [85, 86]. Genetic ablation or pharmacological inhibition of EP1 receptor in mice did not affect the seizure threshold but prevented the likelihood of SE and aggravation of seizure severity, indicating the involvement of EP1 receptor in seizure exacerbation [87, 88]. Unlike EP1, studies on the involvement of EP2 receptor in neuroprotection from seizures had shown quite debatable findings. EP2 antagonism using 3-aryl-acrylamide derivatives attenuated SE-induced neuroinflammation and neuronal injury in rodents [55, 89–91]. Conflictingly, few other studies found systemic administration of EP2 agonists to have significant anticonvulsant effect to PTZ- and pilocarpine-induced seizures [92, 93]. This dual effect of EP2 modulation on neuronal activity depends on the latency to seizure onset and thus shows neuroprotection within a tightly regulated therapeutic window [94, 95]. Such studies question if EP receptors can be considered as potential therapeutic targets in neurological diseases.

Following seizure insults, the developing rat brain showed either no or minor change in COX-2 expression; in contrast, a pronounced increase in COX-2 expression in the adult rat brain was observed [96, 97]. This suggests that COX-2 induction following seizure is age-dependent and the mechanisms regulating its expression and functions are immature in the developing brain [97]. Interestingly, electrically induced SE showed biphasic upregulation of COX-2 in rat hippocampus, an immediate induction at 1 day after SE and induction during spontaneous recurrent seizures (SRS) at the chronic phase, 4–5 months after SE [98]. Moreover, systemic KA administration demonstrated a similar biphasic increase in PG production in the rat hippocampus consisting of an initial burst in the first 30 min and a sustained late-phase production due to COX-2 induction even with a limited AA supply [24]. This suggests that amidst the acute phase and the chronic phase, some recovery mechanisms are activated, which can prevent the exacerbation of seizure-induced neuronal damage, but failed to prevent epileptogenesis.

In respect of human subjects, so far, only four studies have investigated COX-2 expression profile in the hippocampal tissue from patients with DRTLE [19, 98–100] (Table 1). These studies revealed strong immunohistochemical expression of COX-2 in neurons, astrocytes, and microglial cells of hippocampal sclerotic tissue. While Desjardins et al. suggested the implication of COX-2 induction in the pathogenesis of HS in TLE [101], Weidner et al. found no difference in COX-2 expression between TLE with HS and TLE without HS groups implying COX-2 induction to be independent of presence/absence of HS [100]. More recently, epilepsy researchers began working on the relevance of genetic aspects of COX-2 in people experiencing seizures by investigating associated genetic variants [101].

**Table 1:** Clinical studies reporting COX-2 involvement in epilepsy

Reference	Study type	Tissue	Cells	Study subjects	Reference
Desjardins et al. [99]	Expression	Hippocampus	Astrocytes and neurons	5 sclerotic and 2 non-sclerotic DRTLE	Induction of astrocytic COX-2 in patients with HS suggesting its implication in the pathogenesis of HS in epilepsy
Holtman et al. [98]	Expression	Hippocampus	Astrocytes and neurons	6 sclerotic and 4 non-sclerotic DRTLE and 5 controls	Higher astrocytic and neuronal COX-2 in patients with HS compared to non-HS and controls
Das et al. [19]	Expression	Hippocampus	Astrocytes and neurons	6 sclerotic DRTLE and 3 sudden-death controls	Increased COX-2 in patients suggesting its crucial role in TLE pathogenesis
Hung et al. [101]	Genetic	Whole blood	White blood cells	35 children with febrile seizures and 31 controls	A single SNP, rs689466, localized at 5′-1192 of the *PTGS2* gene was significantly association with febrile seizures
Weidner et al. [100]	Expression	Hippocampus	Microglia, astrocytes and neurons	16 sclerotic and 17 non-sclerotic DRTLE	Higher microglial and neuronal COX-2 expression than astrocytic COX-2 No difference in COX-2 levels among sclerotic and non-sclerotic samples

### COX-2-mediated blood-brain barrier disruption

Regulating the trafficking of circulating molecules between the blood and the brain, there exists a selectively permeable monolayer of brain capillary endothelial cells called BBB. Disruption of this barrier is associated with neurological ailments where it can be a cause or appear as a consequence of the disease [102]. Activation of NMDA receptor by the excitatory neurotransmitter, glutamate, disrupted BBB function and permeability in human brain capillary endothelial cells [103] whereas its inhibition by the antagonist, MK-801, prevented BBB breakdown in isolated rat brain capillary endothelial cells [104], suggesting neuronal over-excitation to cause BBB disruption. Several studies demonstrated BBB disruption to be related to seizure occurrence in humans and rodents [105–109]. It was proposed that the resultant disruption leads to BBB leakage, further causing efflux of administered AEDs [110]. Since the brain as well as brain endothelial cells express several well-characterized ABC efflux transporters such as P-glycoprotein (P-gp), BCRP, MRP1, MRP4, and MRP5 [111], these are often associated with AED resistance attributed to BBB leakage in epilepsy [7]. Of these transporters, P-gp has been widely studied for causing multidrug resistance in epilepsy due to its upregulation during seizure. Several investigations revealed upregulation of P-gp in the brain and BBB due to seizure activity in rodent models [112–117] as well as in human patients with refractory epilepsy [117–121]. Rats with drug-resistant seizures were also shown to exhibit enhanced brain P-gp expression compared to those with drug-responsive seizures [122]. Consequently, the prescribed AEDs gets effluxed out into the circulation by the upregulated P-gp, even before reaching the drug target, thereby causing pharmacoresistance.

The underlying molecular mechanism behind the regulation of seizure-induced P-gp is currently being investigated in in vivo and in vitro model systems to achieve the goal of decreasing pharmacoresistance and establishing highly efficacious treatment for epilepsy. P-gp had been observed to be upregulated by glutamate-mediated NMDA receptor activation in brain capillary endothelial cells [123–125]. This glutamate-mediated P-gp upregulation was prevented by exposure to selective COX-2 inhibitors such as celecoxib and NS-398 and non-selective inhibitor, indomethacin heptyl ester [124–126], suggesting the involvement of COX-2 in regulating P-gp expression. Brain capillary endothelial cells from pilocarpine-induced SE rat model also showed high P-gp expression and activity which was blocked by treatment with COX-2 inhibitors, indomethacin and celecoxib [124, 126], revealing the role of COX-2 in seizure-induced P-gp upregulation. Similarly, administration of COX-2 inhibitors, SC-58236 and NS-398, both counteracted the SE-associated increase in P-gp expression in the parahippocampal cortex and the ventral hippocampus in rats [127]. Besides, blocking the EP1 receptor by the antagonist, SC-51089, in pilocarpine-induced SE rat model prevented seizure-induced P-gp upregulation [128]. These investigations indicated that activation of COX-2/EP receptor signaling during seizure is somehow causing upregulation of P-gp, thereby leading to decreased drug delivery to the brain and enhanced resistance to drugs. Direct inhibition of P-gp may improve seizure control; however, its pan inhibition may lead to deleterious effects [129]. Moreover, when a known P-gp inhibitor, verapamil, was administered to pediatric patients, children with drug-resistant epilepsy receiving AED polytherapy showed no significant difference in seizure control compared to those receiving placebo along with AED polytherapy [130]. Likewise, inhibition of multiple EP receptors simultaneously may produce adverse effects. This signifies the importance of targeting the upstream regulatory molecule, COX-2, for a potential future strategy for epilepsy treatment.

### COX-2 inhibitors: therapeutic strategies

Non-steroidal anti-inflammatory drugs (NSAIDs), the currently available COX inhibitory drugs, prevent the formation of PGs by competitively inhibiting the activity of COX enzymes. NSAIDs are of two types: selective, which inhibit only COX-2 (e.g., celecoxib and rofecoxib), and non-selective, which inhibit both COX-1 and COX-2 (e.g., aspirin, ibuprofen, and indomethacin). These inhibitors work by varying degrees of reversible (example, ibuprofen and indomethacin) or irreversible (example, celecoxib, rofecoxib, and aspirin) competitive inhibition. NSAIDs help in mitigating different manifestations of allergic reactions and provide antipyretic, analgesic, and anti-platelet effects in acute and chronic conditions; however, they also have several undesirable effects. Due to the protective role of COX-1 in the maintenance of the stomach lining by preventing it from stomach acid, its inhibition sometimes causes gastric problems. In response to this challenge, new generational NSAIDs, specific to COX-2, were developed. However, the selective COX-2 inhibitors, coxibs, can increase the risk of adverse renal and cardiovascular events and thus require improvisation for better efficacy without any complications.

### COX-2 inhibitors as anticonvulsants

To ascertain the anticonvulsive therapeutic potential of COX-2 inhibition, several studies investigated the effect of COX-2 inhibitors on seizure activity and development in animal models of epilepsy (Table 2). The findings differ with different seizure and treatment conditions. Dhir et al. 2006a examined the effect of selective COX-2 inhibitors, nimesulide and rofecoxib, administered 45 min prior to an epileptic challenge in different mice models [138]. They found that the inhibitors prolonged the mean onset time of convulsions and decreased the seizure duration and the percentage mortality rate against bicuculline- and picrotoxin-induced seizures; however, they did not affect maximal electroshock-induced seizures, suggesting varied efficacy of COX-2 inhibitors in different types of convulsive challenge. Another study by the same group revealed the two selective COX-2 inhibitors to be more efficacious than non-selective COX-2 inhibitors, aspirin and naproxen [140], in antagonizing the effect of PTZ-induced seizures. The authors also found that nimesulide provides a neuroprotective effect by controlling the biochemical alterations caused by PTZ-induced chemical kindling in mice [141]. Administration of 2 mg/kg and 4 mg/kg of rofecoxib increased the seizure threshold; however, a lower dose of 1 mg/kg of rofecoxib failed to do the same, indicating a dose-dependent effect [145]. Pretreatment of selective COX-2 inhibitor, celecoxib, 60 min prior to seizure induction also demonstrated anticonvulsant effects in the PTZ-induced rat model [84]. Conversely, several studies suggest that pretreatment of selective or non-selective COX-2 inhibitors may have an inverse effect by acting as proconvulsants [132–134, 139, 151], and a postictal COX-2 inhibition would rather have a neuroprotective effect [132, 142, 144, 149, 150]. After PTZ challenge to male Wistar rats, the selective COX-2 inhibitor, etoricoxib, showed an anticonvulsant effect at a dose of 1 mg/kg which got reduced or reversed at 10 mg/kg of dose displaying the neuroprotective effect within a narrow therapeutic dose window [137].

**Table 2:** Preclinical evidences supporting or opposing clinical application of COX-2 inhibitors for epilepsy treatment

Selectivity	Drug	Type of convulsive challenge	Supporting evidences	Opposing evidences
Selective	Celecoxib	Electrical stimulation	[131]	[87]
		Flurothyl	[96]	–
		Kainic acid	[132]	[133, 134]
		Pentylenetetrazol	[84]	–
		Pilocarpine	[126, 135]	–
	Etoricoxib	Genetic model	[136]	–
		Pentylenetetrazol	[137]	–
	Nimesulide	Bicuculline	[138]	–
		Electrical stimulation	[6, 23]	–
		Kainic acid	–	[134, 139]
		Pentylenetetrazol	[140, 141]	–
		Picrotoxin	[138]	–
	NS-398	Kainic acid	[83]	[133]
		Pilocarpine	[127, 142]	–
	Parecoxib	Pilocarpine	[143]	–
	Rofecoxib	Kainic acid	[144]	–
		Pentylenetetrazol	[56, 140, 145]	[146]
	SC-58125	Kainic acid	[82]	–
	SC-58236	Electrical stimulation	–	[98, 147]
		Pilocarpine	[127]	–
Non-selective	Aspirin	Electrical stimulation	[148]	–
		Kainic acid	–	[133]
		Pilocarpine	[149, 150]	[151]
	Ibuprofen	Electrical stimulation	[148]	–
	Indomethacin	Electrical stimulation	[148]	–
		Kainic acid	–	[133, 151]
	Others (metamizole, paracetamol, piroxicam, ketoprofen)	Electrical stimulation	[148]	–
		Kainic acid	–	[134]

**Fig. 2:**
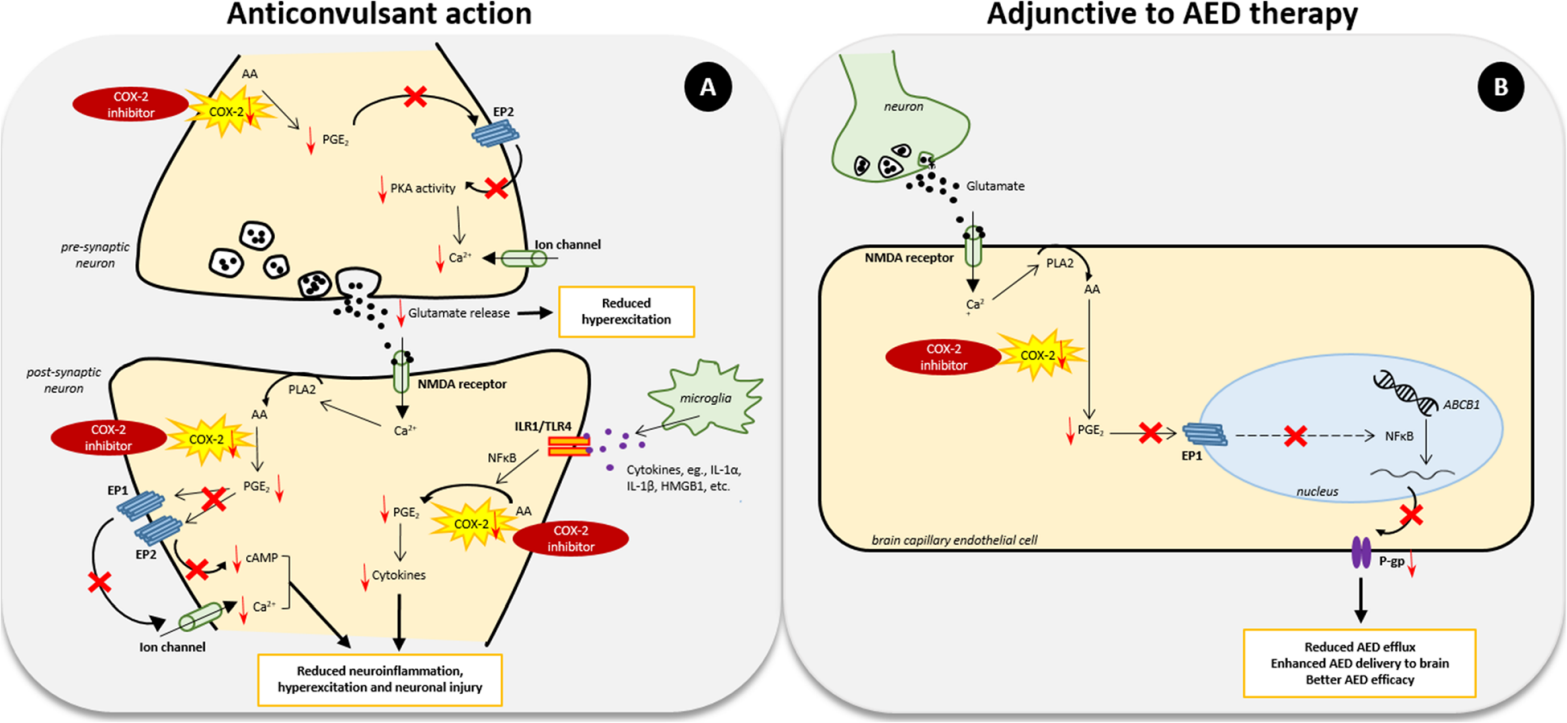
Clinical use of COX-2 inhibitors in epilepsy treatment. **a** Anticonvulsant, COX-2 inhibitors reduce the production of PGE_2_ causing decreased activation of EP receptors which, in turn, lowers calcium ion influx and release of glutamate, thus blocking the seizures. They also reduce neuroinflammation by decreasing the production of cytokines in the brain cells. **b** Adjunctive to AED therapy, COX-2 inhibitors reduce activation of EP1 receptor by decreasing the production of PGE_2_ which, in turn, follows an unknown cascade of biological events leading to downregulation of the efflux transporter, P-glycoprotein, at the blood-brain barrier. This ultimately results in reduced efflux of the administered AED/s, further enhancing their brain uptake and hence efficacy

Celecoxib administration 1 day after pilocarpine-induced SE reduced the likelihood of developing SRS and prevented hippocampal neuronal damage in rats [135]. Early long-term treatment of another coxib drug, etoricoxib, also displayed antiepileptogenic effect by reducing the development of absence seizures in the genetic WAG/Rij rat model of absence epilepsy [136]. However, several reports contradict these findings. Administration of rofecoxib 5 days prior to epileptic challenge showed no effect on PTZ-kindling development [146]. A 3-day treatment with the selective COX-2 inhibitor, SC-58326, starting 1 day before electrically induced SE increased rat mortality in models of TLE [147] while a 7-day treatment starting 4 h after SE induction effectively reduced PGE_2_ production but did not prevent seizure development or neuronal damage [98]. Similarly, an 18-day administration of the selective COX-2 inhibitor, parecoxib, following pilocarpine-induced SE prevented the subsequent increase in PGE_2_ and reduced seizure severity in the rat hippocampus and piriform cortex; however, it could not prevent the development, frequency, and duration of seizures [143]. These studies demonstrate an anticonvulsive but not antiepileptogenic effect of COX-2 inhibitors. It is proposed that COX-2 inhibitors display this anticonvulsive activity by reducing the production of PGE_2_ causing decreased activation of EP receptors which, in turn, lowers calcium ion influx and release of the excitatory neurotransmitter, glutamate, thus blocking the seizures [152] (Fig. 2a). Simultaneously, COX-2 inhibitors suppress the production of pro-inflammatory cytokines reducing inflammation [72–74].

In vivo studies reporting the effect of COX-2 inhibitors on seizure activity revealed that various factors determine the anticonvulsant action of these drugs viz., dosage, time of administration, treatment duration, type of convulsive challenge, and selectivity of COX-2 inhibitors. Based on these factors, COX-2 inhibitors, alike EP receptors, can produce dichotomous effects, neuroprotective or neurotoxic [153], suggesting the need to optimize their therapeutic dose, time, treatment duration, and other pharmacokinetic and pharmacodynamic properties. However, instead of targeting multiple EP receptors, targeting the upstream COX-2 enzyme would produce a broad-spectrum effect and therefore remains the key therapeutic target for epilepsy treatment.

### COX-2 inhibitors as adjunctive to AED therapy

As discussed earlier, COX-2 may regulate the expression of the multidrug transporter, P-gp, which is found to be overexpressed in drug-resistant epilepsy. COX-2 upregulation following seizure produces higher levels of PGE_2_, which when bound to the EP1 receptor, activates a signaling cascade involving the transcription factor, NF-κB, leading to increased expression of P-gp [7]. Overexpression of P-gp at the BBB may result in enhanced the efflux of the prescribed AED/s even before reaching the target site in the brain, causing pharmacoresistance. Blocking seizure-mediated P-gp overexpression in the brain capillary endothelial cells, therefore, may facilitate better drug delivery to the brain and improve drug efficacy provided the prescribed AED is a substrate of P-gp. Several studies have investigated different AEDs as potential substrates of P-gp. Phenytoin, phenobarbital, lamotrigine, and levetiracetam have widely been observed to be weak but potential substrates of P-gp while carbamazepine, felbamate, and ethosuximide had no substrate interaction with the transporter [154]. Other drugs have shown either contradictory findings or remain insufficiently investigated in this regard.

Overexpression of P-gp at the BBB decreased brain uptake of phenytoin at specific limbic brain regions of chronic epileptic rats [155]. Its direct inhibition using three different P-gp inhibitors resulted in increased phenytoin concentration in brain extracellular fluid (ECF) in rats [156]. Treatment with selective P-gp inhibitor, tariquidar, improved seizure control in chronic epileptic rats due to increased phenytoin delivery to the brain [155, 157]. Despite these beneficial findings of direct P-gp inhibition, its pan inhibition may lead to deleterious effect [129], thus an alternative indirect approach is required for its suppression. Since COX-2 serves as a transcriptional regulator of P-gp, inhibiting COX-2 may assist in achieving enhanced efficacy of prescribed AEDs (Fig. 2b). In vitro data revealed prevention of glutamate-induced P-gp upregulation by selective and non-selective COX-2 inhibitors in rodent and human brain capillary endothelial cells [124–126]. COX-2 inhibitors also blocked SE-induced P-gp upregulation in the brain capillaries of epileptic rats, revealing the role of COX-2 inhibitors in preventing seizure-induced P-gp upregulation [124, 126]. Furthermore, brain uptake of phenytoin was significantly enhanced by sub-chronic COX-2 inhibition via suppressing P-gp expression in chronic epileptic rats, suggesting that COX-2 inhibitors may help in increasing drug delivery to the target sites in the brain [127]. To relate this effect of COX-2 inhibitors with the drug efficacy, Schlichtiger et al. investigated the outcome of celecoxib treatment in phenobarbital-treated responder and non-responder epileptic rats [131]. A 6-day treatment with celecoxib significantly reduced P-gp expression as well as frequency of SRS in both the responders and non-responders suggesting the potential role of COX-2 inhibition in increasing AED efficacy via downregulating P-gp. Rofecoxib also potentiated the anticonvulsant activity of the AED, tiagabine, against PTZ-induced seizures in mice [56]. Non-selective NSAIDs such aspirin, ibuprofen, indomethacin, metamizole, paracetamol, and piroxicam also enhanced the anticonvulsive activity of valproate while the anticonvulsive activity of phenytoin was increased only by ibuprofen and piroxicam against maximal electroshock-induced seizures in mice [148]. Therefore, besides their anticonvulsive effect, COX-2 inhibitors also show a great promise towards being an adjunctive therapy for improving the efficacy of administered AEDs.

#### Human clinical studies

Despite decades of extensive research on the beneficial role of COX-2 inhibition in controlling seizure and drug-resistance in epilepsy, the selective COX-2 inhibitors have, so far, not been clinically tested in patients with epilepsy due to their severe adverse effects. However, a recent report by Lim et al. [158] investigated the effect of celecoxib on the neuronal excitability and electrophysiological properties of the brain of healthy volunteers. Though the authors found no effect of celecoxib on the neuronal excitability of the healthy volunteers, the study is limited due to the absence of inflammation in the healthy subjects to observe the effect of the anti-inflammatory drug, celecoxib. In regard to the non-selective COX-2 inhibitors, aspirin, having relatively fewer side effects, has been investigated independently or in adjunction to AED therapy for controlling seizures in epilepsy and related syndromes (Table 3) to substantiate the findings of preclinical studies reporting efficient seizure reduction upon administration of COX-2 inhibitors (Table 2). Most of these studies were performed on patients with the rare neurological disorder, Sturge-Weber syndrome, limiting the current knowledge on the applicability of these drugs for other seizure disorders or epilepsy. Long-term continuous use of aspirin in patients with Sturge-Weber syndrome resulted in seizure freedom for at least 1 year in five of six patients [160]. An internet-based survey involving patients with Sturge-Weber syndrome receiving aspirin reported seizure reduction in 21 of 34 patients [161], suggesting the use of low-dose aspirin to be safe and beneficial. The findings were further validated by Lance et al. who observed seizure control in 91% of the patients with the syndrome receiving low-dose aspirin with minimal side effects [162]. Patients with focal epilepsy receiving aspirin also showed significantly fewer seizures compared to age-, sex- and disease-matched controls not receiving aspirin [163]. They found an inverse correlation between aspirin doses and seizure frequency. However, a randomized, double-blind, placebo-controlled trial failed to demonstrate a preventive effect of another non-selective NSAID, ibuprofen, on the number of febrile seizure recurrences in 230 children at increased risk [159] (Table 3). Therefore, studies revealing the anticonvulsive effect of aspirin require replication in a large-sample, randomized, controlled trial to substantiate the effect and applicability of COX-2 inhibitors as a therapeutic approach in epilepsy management.

**Table 3:** Clinical evidences using non-selective NSAIDs in patients experiencing seizures

Reference	NSAID	Use	Study subjects	Effects
van Stuijvenberg et al. [159]	Ibuprofen	Independent	Randomized, double-blind, placebo-controlled study in 230 children with febrile seizures (111 on ibuprofen and 119 on placebo)	Failed to reduce number of seizure recurrences in children at increased risk
Udani et al. [160]	Aspirin	Not reported	9 children with Sturge-Weber syndrome (6 with long-term continuous aspirin therapy and 3 with intermittent use of aspirin)	Seizure freedom for at least one year in 8 of 9 children
Bay et al. [161]	Aspirin	In adjunction to AED therapy	Internet-based survey in 34 subjects with Sturge-Weber syndrome receiving aspirin	Seizure reduction in 21 of 34 patients
Lance et al. [162]	Aspirin	In adjunction to AED therapy	58 subjects with Sturge-Weber syndrome receiving aspirin	Seizure control in 91% of the patients
Godfred et al. [163]	Aspirin	Independent	46 subjects with focal epilepsy (23 receiving aspirin and 23 not receiving aspirin)	Fewer seizures in patients on aspirin therapy than patients not receiving it

## Conclusion

Increasing evidences on the role of inflammation in epilepsy pathogenesis are encouraging researchers to gather information on the clinical use of neuroinflammation-targeted therapeutics and to develop their better, improved, efficacious analogues. COX-2, a proinflammatory enzyme interconnecting various inflammatory processes, is widely being investigated as a therapeutic target in epilepsy. Several preclinical and clinical studies demonstrated COX-2 induction during a seizure event and in epilepsy. Pharmacological inhibition of COX-2 enzyme using selective and non-selective COX-2 inhibitors not only resulted in reduced seizure recurrence and disease severity but also increased the efficacy of administered AEDs, suggesting their two probable modes of action, (1) anticonvulsive and (2) adjunctive to AED therapy. However, the efficacy of COX-2 inhibitors depends on various factors such as viz., therapeutic dose, time of administration, treatment duration, and their selectivity and is often accompanied by mild or severe adverse effects, thus prompting further investigations on improvising the efficacy and optimizing their use without any complications. In addition, future studies should also focus on investigating the anticonvulsive effect of COX-2 inhibitors in large-sample, randomized, controlled trials to substantiate the clinical application of COX-2 inhibitors as a future therapeutic strategy for epilepsy management.

## Data Availability

Not applicable.

## Abbreviations

AA: Arachidonic acid
ABC: ATP-binding cassette
AED: Antiepileptic drug
BBB: Blood-brain barrier
CNS: Central nervous system
COX: Cyclooxygenase
CSF: Cerebrospinal fluid
DRTLE: Drug-resistant temporal lobe epilepsy
ECF: Extracellular fluid
GABA: γ-Aminobutyric acid
GPCR: G protein-coupled receptor
HS: Hippocampal sclerosis
KA: Kainic acid
LPS: Lipopolysaccharide
NMDA: N-Methyl-d-aspartate
NO: Nitric oxide
NSAID: Non-steroidal anti-inflammatory drug
PG: Prostaglandin
P-gp: P-glycoprotein
PTZ: Pentylenetetrazol
SE: Status epilepticus
TLE: Temporal lobe epilepsy
